# The role of DGAT1 and DGAT2 in regulating tumor cell growth and their potential clinical implications

**DOI:** 10.1186/s12967-024-05084-z

**Published:** 2024-03-18

**Authors:** Boer Deng, Weimin Kong, Xiaochang Shen, Chao Han, Ziyi Zhao, Shuning Chen, Chunxiao Zhou, Victoria Bae-Jump

**Affiliations:** 1 Department of Gynecologic Oncology, Beijing Obstetrics and Gynecology Hospital, Capital Medical University, Beijing Maternal and Child Health Care Hospital, Beijing, People’s Republic of China; 2 Division of Gynecologic Oncology, University of North Carolina at Chapel Hill, Chapel Hill, NC 27599 USA; 3 Lineberger Comprehensive Cancer Center, University of North Carolina at Chapel Hill, Chapel Hill, NC 27599 USA

**Keywords:** Diacylglycerol acyltransferase, Lipid droplet, Tumor growth, Prognosis

## Abstract

Lipid metabolism is widely reprogrammed in tumor cells. Lipid droplet is a common organelle existing in most mammal cells, and its complex and dynamic functions in maintaining redox and metabolic balance, regulating endoplasmic reticulum stress, modulating chemoresistance, and providing essential biomolecules and ATP have been well established in tumor cells. The balance between lipid droplet formation and catabolism is critical to maintaining energy metabolism in tumor cells, while the process of energy metabolism affects various functions essential for tumor growth. The imbalance of synthesis and catabolism of fatty acids in tumor cells leads to the alteration of lipid droplet content in tumor cells. Diacylglycerol acyltransferase 1 and diacylglycerol acyltransferase 2, the enzymes that catalyze the final step of triglyceride synthesis, participate in the formation of lipid droplets in tumor cells and in the regulation of cell proliferation, migration and invasion, chemoresistance, and prognosis in tumor. Several diacylglycerol acyltransferase 1 and diacylglycerol acyltransferase 2 inhibitors have been developed over the past decade and have shown anti-tumor effects in preclinical tumor models and improvement of metabolism in clinical trials. In this review, we highlight key features of fatty acid metabolism and different paradigms of diacylglycerol acyltransferase 1 and diacylglycerol acyltransferase 2 activities on cell proliferation, migration, chemoresistance, and prognosis in tumor, with the hope that these scientific findings will have potential clinical implications.

## Background

Cancer cells have the capacity to change their metabolism including upregulation of glycolysis, amplified de novo fatty acid synthesis, increased glutaminolysis and biosynthetic and bioenergetic pathways in order to meet the higher energy demands [1–5]. These processes of changing metabolism, also known as metabolic reprogramming, create a favorable environment for cell proliferation, invasion, metastasis, and chemotherapy resistance [6]. Although most cancer cells rely on glycolysis as an energy source, cancer cells have a greater ability to acquire extracellular lipids and have elevated lipid metabolism such as de novo lipogenesis, fatty acid (FA) uptake, and FA oxidation (FAO) for energy production, lipid accumulation, signal transduction intermediates and plasma membrane synthesis [7, 8]. Irregularities in the synthesis and catabolism of lipid may lead to abnormal accumulation of FAs in cancer cells, resulting in cell damage (so called lipotoxicity) through processes like death receptor activation, endoplasmic reticulum stress, mitochondrial activity changes, and oxidative stress [9, 10]. Maintaining energy homeostasis for self-protection by accumulating energy-rich neutral lipids in the form of FAs in specialized organelles called lipid droplets (LDs) is crucial for the survival of cancer cells [2, 11]. Most normal cells have a restricted ability to retain lipids, and if the processes that break down lipids exceed the capacity to convert intracellular free FAs into esters, excess free FAs within the cells can cause harmful effects such as cytotoxicity, ectopic storage, and susceptibility to lipotoxicity [12]. In contrast, cancer cells have a remarkable capacity to store lipids in the form of LDs, which protects them from the harmful effects of excess free FAs while they continue to synthesize and catabolism high levels of FAs [13, 14].

**Fig. 1:**
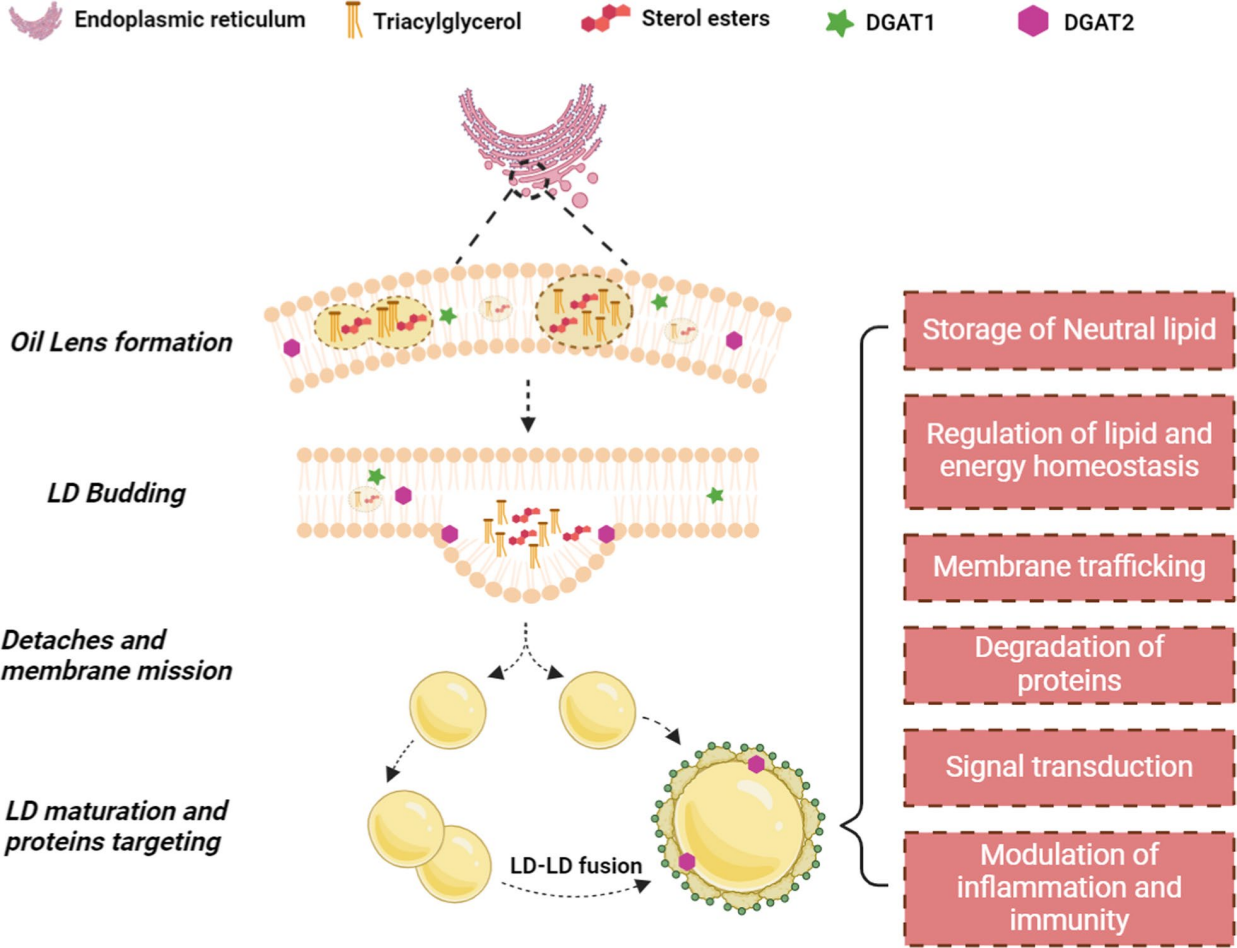
Biogenesis and functions of Lipid droplets. The biogenesis of Lipid droplets (LDs) in normal and tumor cells is a complex process, consisting of multiple steps, briefly including neutral lipid synthesis and lens formation, lipid droplet bidding, lipid droplet growth and maturation and protein targeting to lipid droplets [19]. The whole process of LD biogenesis occurs in the endoplasmic reticulum (ER) and the essential enzymes catalyzed synthesis of neutral lipids, including diacylglycerol acyltrans-ferases (DGATs) and acyl-CoA:cholesterol O-acyltransferases (ACATs) also located in the ER [19]. The first step is synthesis of neutral lipids (triacylglycerols and sterol esters) between the leaflets of the ER bilayer [21]. Accompanied by the deposit and incrased concentration of neutral lipids, an oil lens is formated in a process of demixing when the concentration of triacylglycerol is in the range of 5–10 mol% [19]. Under the regulation of ER membrane phospholipid and membrane surface tension, the LD budding is observed as a result of expansion of the neutral lipid lens. Through droplet–droplet fusion, LDs expand and a population of LDs eventually detaches from the ER in higher eukaryotes. Upon fusion, integral membrane proteins such as DGAT2, are able to diffuse from the ER to LDs, whereas the DGAT1 only exists in ER. Finally, up to 100–200 proteins, dominated by enzymes involved in lipid metabolism and members of the peri-lipin family target to LDs to endow LDs with dynamic and complex functions [15]

LDs exist in nearly all human cells and are the major formation of lipid storage, first discovered by van Leeuwenhoek in 1674 and reported in 1963 as an organelle derived from the endoplasmic reticulum (ER) [3, 15, 16]. LDs were previously thought of as inert fat droplets with few functions but neutral lipid storage, but more recently LDs have been shown to play complex and dynamic roles in the regulation of lipid metabolism and energy homeostasis, membrane synthesis, turnover and degradation of proteins, signal transduction, and modulation of inflammation and immunity through communication with other organelles [17–20]. The biogenesis and functions of LDs have been extensively discussed in previous literature (Fig. 1), but there are still many unknown biological processes and functions need to be further studied with more advanced detection technology [13, 19, 21]. The intracellular content of LDs reflects the balance between lipid synthesis and lipid consumption, and excessive accumulation of LDs mainly occurs in fat-related diseases, such as obesity, nonalcoholic steatohepatitis, and arteriosclerosis [16]. Recently, LDs have been identified as a prominent characteristic associated with carcinogenesis, progression, and chemoresistance in multiple types of cancer [3]. Importantly, LDs have also been shown to promote cancer stem cell (CSC) phenotypes that contribute to tumor cell renewal and resistance to chemotherapy or radiotherapy [3, 22]. The regulation of LD formation in tumor tissues is a complex process entailing numerous biological processes, including inflammation, hypoxia, and acidosis, along with the dysregulation of phosphatase and tensin homolog deleted on chromosome ten (PTEN), kirsten rat sarcoma viral oncogene (KRAS), and forkhead Box Protein O3 (FOXO3)/Sirtuin6 signaling pathways [3, 23–26].

Diacylglycerol acyltransferase (DGAT) isoform enzymes, consisting of DGAT1 and DGAT2, are responsible for the last step of TG synthesis, converting the diacylglyceride (DAG) to TG and contributing to the formation of LDs in a variety of human cells. DGAT1 and DGAT2 are involved in the maintenance of lipid metabolism homeostasis in benign and neoplastic cells, and emerging evidence suggests that the expression levels of these two isoforms vary in different tissues and organs [27, 28]. In mammal cells, DGAT1 and DGAT2 ubiquitously express in most tissues with different relative levels of expression. DGAT1 is most highly expressed in the small intestine, adrenal gland, rectum and duodenum tissue, and its activity is essential for the absorption of dietary fat through acylation of acylglycerols in the intestine, which is accountable for the esterification of exogenous fatty acids (FAs) to glycerol and is required for the synthesis of fat for storage in almost all human somatic cells and tumor cells [27]. DGAT2 is primarily expressed in liver, adipose, mammary gland, testis, peripheral leukocytes, and cardiac tissues, and is an enzyme necessary for the synthesis and storage of intracellular lipids [26, 28, 29]. Overexpression of each isozyme contributes to increased TG synthesis and accumulation of LDs, while inhibiting their activities significantly reduces the synthesis of TGs and the density of LDs in normal cells and tumor cells [30]. Mice with DGAT1 deficiency are lean and resistant to diet-induced obesity, while DGAT2 deficiency results in reduction of the majority of fat in the whole body [31, 32]. A specific DGAT1 inhibitor, Yhhu2407, significantly reduced plasma TG in mice and exerted a beneficial effect on regulating lipoprotein levels in rats [33]. Inhibition of DGAT2 activity by anti-sense oligonucleotides effectively reduced hepatic lipid (DAG and TG) levels and improved insulin sensitivity in rats, indicating a potent strategy for weight loss and metabolic regulation by targeting DGAT2 [34]. Based on such metabolic regulatory benefits, DGAT1 or DGAT2 inhibitors, including PF-04620110 and Ervogastat (PF-06865571), have already been advanced to human Phase I-II clinical trials in healthy adults and individuals with non-alcoholic steatohepatitis or diabetes and demonstrated a significant reduction in postprandial TGs and liver fat percentage. In addition, a DGAT1 inhibitor (pradigastat) markedly reduced fasting TG level in patients with familial chylomicronemia syndrome in a phase III clinical trial [35].

Since disruption of aberrant lipid metabolism and subsequent LD accumulation are implicated in carcinogenesis and tumor progression, the overexpression of DGAT1 and DGAT2 in a variety of tumor cells and their regulatory roles in the formation of LDs and in the control of tumor cell growth have generated interest as to whether inhibiting DGATs can affect the growth of tumor cells [3, 23, 35–39]. In this review, we focus on uncovering the relationship of DGAT1 and DGAT2 activities and LD formation in regulating cell proliferation, apoptosis, invasion, the anti-tumor immune response, chemoresistance, and radioresistance of tumor cells, in an attempt to gain a deeper understanding of DGATs and tumor growth, as well as their potential clinical implications for cancer patients.

## Elevated FAs uptake and de novo lipogenesis endow tumor cells with sufficient FA sources

The infinite proliferative and metastatic activities of tumors dictate that tumor cells have much higher energy requirements than benign cells, making them more susceptible to harsh nutritional conditions [40]. Lipids, acting as a compensatory mechanism for tumor cells in the face of metabolic stress and decreased glycolysis, become important energy sources for tumor cells to meet their high adenosine triphosphate (ATP) demands [41]. Reprogramming of FA metabolism in tumor cells involves the uptake of exogenous FAs, de novo lipogenesis (DNL), β-oxidation of FAs (FAO), and storage of FAs as TGs. Tumor cells acquire FAs through uptake of exogenous FAs, DNL (endogenous FAs), and lipolysis of TGs in LDs intracellular, and FAs from these approaches contribute to the cellular FAs pool of tumor cells [41]. Very low-density lipoproteins and nascent chylomicrons secreted by the liver and intestine are the main forms of plasma TGs and act as the main source of exogenous lipids for tumor cells [42, 43]. Uptake of exogenous FAs into tumor cells primarily occurs through the process of fatty acid endocytosis, mediated by specialized transporters, including the fatty acid translocase/CD36, fatty acid transport protein family, and the plasma membrane fatty acid-binding proteins [44]. Compared to benign cells, tumor cells upregulate the expression levels of these proteins to facilitate efficient transfer of exogeneous FAs across the plasma membrane, thereby providing more lipid molecules, ATP, and nicotinamide adenine dinucleotide phosphate (NADPH) for tumor growth and facilitating lipid storage in LDs in tumor cells [19].

DNL is a complex and highly regulated process in which excess alternative circulation carbohydrates (glucose, glutamine, and acetate) are converted into FAs to synthesize either TGs or other lipid molecules [45, 46]. Physiologically, metabolically active hepatocytes and adipocytes have a high activity of DNL to meet their metabolic needs and energy homeostasis [46]. Most tumor tissues and their precursor lesions unexpectedly undergo exacerbated DNL [47]. Upregulation of a series of coordinated lipogenic enzymes, such as ATP-citrate lyase (ACLY), acetyl-CoA carboxylase (ACC), fatty acid synthase (FASN), and stearoyl-CoA desaturase-1 (SCD1), endows potent lipogenic capacity on tumor cells, and changes in these enzymes have been found at different stages of tumorigenesis and tumor progression [46, 47]. Excess pyruvate produced by high glycolytic activity of tumor cells is an important substrate for DNL [48, 49]. By increasing the DNL, tumor cells can generate a diverse cellular pool of lipid species, including palmitate (FA16:0), stearate (FA18:0), oleate (FA18:1), and some complex long-chain FAs, which allow tumor cells to better adapt to different microenvironments and to better resist chemotherapy and radiotherapy [40, 41, 50–52]. Tumor cells appear to maintain high levels of endogenous FAs synthesis, regardless of whether intracellular FA levels exceed tumor cell requirements [40, 53–56]. FAs in the cytoplasm are covalently modified under the catalysis of acyl-CoA synthetases (ACSs) to generate FA acyl-CoA esters that enter the bioactive pool to participate in the subsequent series of anabolism and catabolism [57–60].

Given the frequently upregulated uptake and DNL in tumor cells, tumor cells possess more abundant and diverse FA pools than normal cells, which provide sufficient substrates for their increased FAO and synthesis of TGs [11]. Fatty acid synthesis (FAS) and FAO are two opposite processes that were previously considered to be mutually exclusive reactions that cannot simultaneously co-exist in benign and tumor cells [61]. ACC, including ACC-alpha (also termed ACC1) and ACC-beta (also known as ACC2) located at cytosol and the outer mitochondrial membrane, respectively, is responsible for the regulation of anabolism or catabolism of FAs, depending on intracellular acetyl-CoA and malonyl-CoA levels [61, 62]. Since malonyl-CoA is the precursor of FAS and a potent inhibitor of carnitine palmityl transferase 1 (CPT1), it is suggested that the interplay of FAO and FAS is regulated by ACC, which presents a ‘one-way street’ property in lipid metabolism [63–65]. Recent studies demonstrated that the distinctive localization-dependent compartmentalization of ACC1 and ACC2 may allow simultaneous and independent activation of both FAS and FAO pathways in tumor cells [64, 66]. In addition, FAO metabolism contributes to the accumulation of acetyl-CoA in the cytoplasm that is required for the initiation of FAS, making it possible for tumor cells to maintain high levels of both FAO and FAS [61, 67]. These changes in lipid metabolism of tumor cells interact to constitute a huge metabolism structure of reprogramming FAs in tumor cells, which are involved in the production of ATP and biomolecules and storage as LDs (Fig. 1) [5, 7, 8].

## Excess FAs store as TGs in LDs in tumor cells

**Fig. 2:**
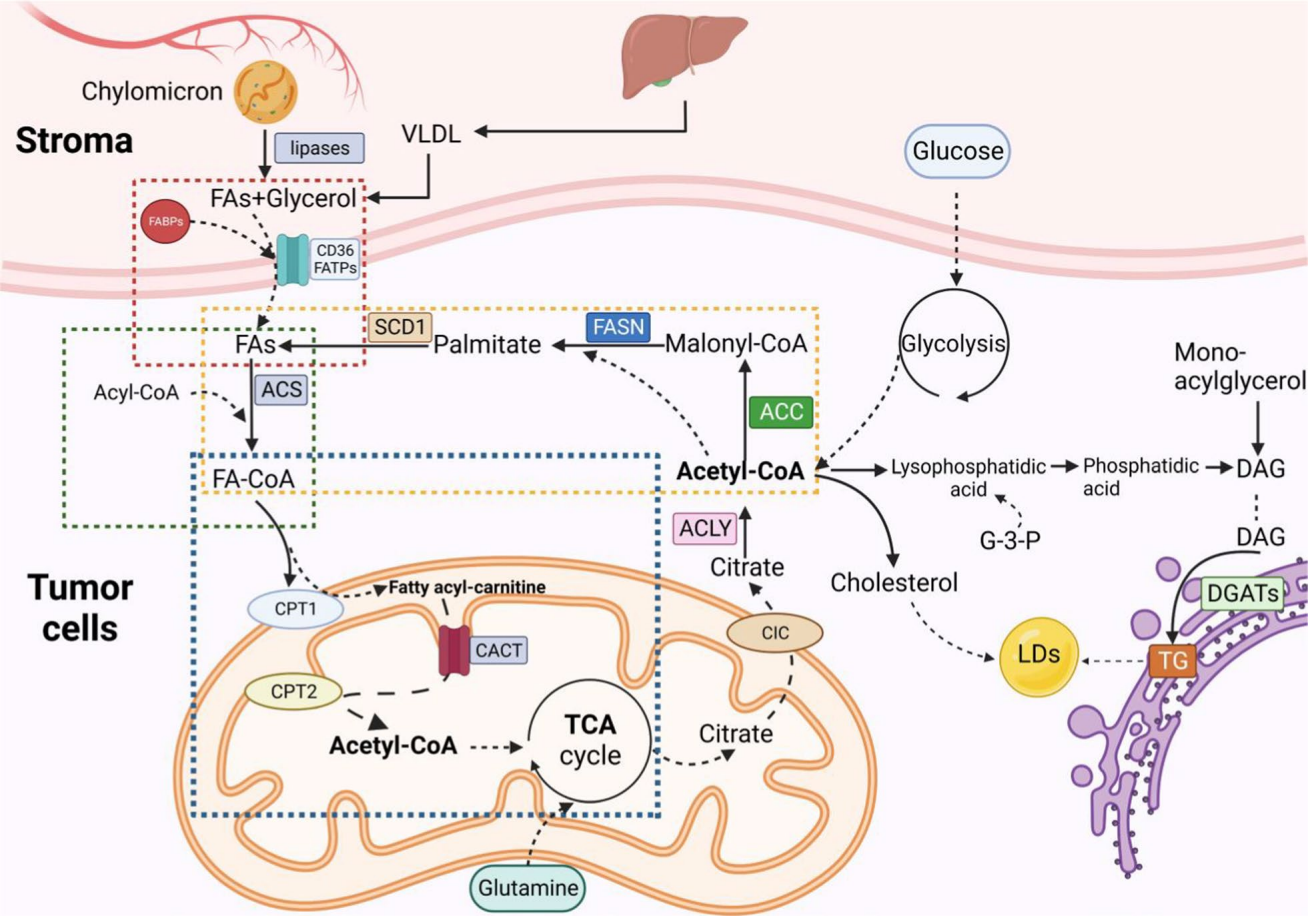
Overview of fatty acid metabolism in tumor cells. Tumor cells reprogram their lipid metabolism, impacting FA uptake (red- dotted line frame), de novo lipogenensis (DNL) (orange-dotted line frame), and activation (green- dotted line frame) of FAs and FAs oxidation (FAO) (blue-dotted line frame). The uptake of exogenous FAs in cancer cells mainly occurs through the process of fatty acid endocytosis, mediated by specialized transporters, including fatty acid translocase (FAT)/CD36, fatty acid transport protein family (FATPs) and plasma membrane fatty acid-binding proteins (FABPpm) [38]. DNL is a crucial component in cancer cell metabolism because of its importance in connecting glucose metabolism and lipid metabolism by catalyzing Acetyl-CoA, a product in glucose metabolism, to synthesis FAs [40, 41]. FAs in the cytoplasm are covalently modified under the catalysis of acyl-CoA synthetases (ACSs) to generate FA acyl-CoA esters that enter the bioactive pool to participate in the subsequent series of anabolism, catabolism, and oxidation. After activation in cytoplasm, FA acyl-CoA moves to the mitochondria to enter fatty acid β-oxidation (FAO), which is a multi-step reaction that involves various enzymes [60]

To inhibit the potential damage and toxicity of deregulated lipids, excess FAs in tumor cells are esterified to glycerol-3-phosphate (G3P) and cholesterol to generate TGs or cholesteryl esters stored in LDs [68]. There are two major pathways of TG synthesis in benign and tumor cells: the G3P pathway and the monoacylglycerol (MAG) pathway, both of which are both catalyzed by DGATs (Fig. 2) [69, 70]. TGs are synthesized and then imported into cytosolic LDs or nascent lipoproteins from TG secreting cells such as hepatocytes [71]. After synthesis, TGs are dispersed between the leaflets of the ER bilayer at low concentrations. When the concentration of TGs is in the range of 5–10 mol%, TGs coalesce and form an oil lens, an expansion of which results in the LD budding from the ER membrane [19]. Although tumor cells are limited in storing energy in the form of carbohydrates, their ability to store TGs appears to be unlimited, even in the presence of abundant amounts of circulating exogenous FAs [40, 46, 72]. Depending on the organism, cell type, and nutrient availability, LDs vary in size from approximately 0.2 to 100 um and consist of a monolayer of phospholipids and a hydrophobic lipid core [73, 74]. The neutral lipid core in LDs consists of up to 100 types of neutral lipids, including TG and steryl esters (SEs), with a range of different FA side chains [75]. The results of proteomic analyses show that more than 200 proteins have been isolated from LDs, which are divided into four categories: structural proteins (perilipin (PLIN) family), membrane transport proteins (e.g., Arf1, SNARE, Rab10), enzymes (neutral lipases), and other LD proteins [3]. Aggregation of these resident proteins localizes to sites of LDs, regulating LD biogenesis and determining the complex and highly dynamic functions of LDs [75, 76]. During periods of high-energy availability, excess intracellular FAs are synthesized as neutral lipids (TGs and SEs) and stored in LDs, while LDs start to break down when the energy level drops [77]. In addition to energy storage, LDs widely participate in a range of other biologic processes, including the storage of hydrophobic vitamins and signaling precursors, management of ER and oxidative stress, protein degradation, and membrane synthesis [14, 17, 78, 79]. Recently, the protective role of LDs against intracellular lipotoxicity, maintenance redox balance, and regulation of autophagy in tumor cells have also been highlighted [77]. Accumulation of LDs in tumor cells is regarded as a critical promoter for carcinogenesis and cancer progression due to its dynamic function in providing energy and phospholipids for cell proliferation and metastasis, minimizing stress and lipotoxicity, and increasing the resistance of tumor cells to chemotherapy and radiotherapy [3, 80–84].

It is currently believed that two distinct populations of LDs coexist within benign and tumor cells, termed growing LDs (smaller LDs resulting from ER budding with the catalyzation of DGAT1) and expanding LDs (larger LDs resulting from the in situ enzymatic activity of DGAT2 that translocate to the LDs from the ER), respectively [73, 85, 86]. The budding of the nascent LD occurs at unique membrane subdomains of ER, and those newly formed LDs are in contact with the ER or are, alternatively, completely released [85, 87]. With the catalysis of DGAT2, additional TG is synthesized locally and added to the neutral lipids core to form expanding LDs [85]. It was proposed that overexpression of DGAT1 resulted in accumulation of small LDs around the cell periphery, whereas overexpression of DGAT2 contributed to increased large LDs intracellularly in rat hepatoma cells [88]. Likewise, a DGAT1 inhibitor (T-863 or PF-04620110) significantly decreased the total number of small and large LDs in breast cancer MCF-7 cells, suggesting that DGAT1 and DGAT2 interact in the process of LD formation in tumor cells [35]. While the functions of these two LDs within individual cells are still under debate, these morphologically distinct populations appear to serve distinct metabolic purposes [75]. Recently, LDs have been discovered to alternatively degrade through lipophagy, a special autophagy that is catalyzed by acid lipases (LIPA/LAL) in acid pH inside lysosomes [68, 80, 89]. In tumor cells, multiple autophagy-related proteins, such as Atg2A and Atg14L, are localized at LDs and participate in the formation of early autophagosomal membranes to facilitate the high lipid turnover, tumor cells tumorigenesis, and metastasis [14, 90, 91].

Increased accumulation of LDs in tumor cells involves several signaling pathways, such as activation of the epidermal growth factor receptor and PI3K/AKT/ mammalian target of the rapamycin (mTOR) pathways and inactivation of the FOXO3/SIRT6 pathway [24]. Other intracellular stimulations, including lipid overload, ER stress, hypoxia, acidic environment, mitochondrial damage, imbalances in energy metabolism and redox homeostasis, and treatment with chemotherapeutics are also reported to trigger the biosynthesis of LDs in tumor cells [84, 92–94]. Notably, cancer-associated fibroblasts (CAFs), an important component of the tumor microenvironment, also participate in the accumulation of LDs by upregulating the secretion of lactate, which subsequently provides acetyl moieties for histone acetylation and establishes a regulatory loop between lipid metabolism and epigenetic modification in prostate tumor cells. Further inhibition of the bromodomain and extra-terminal protein family of histone acetylation readers suppressed the expression of perilipin 2 (PLIN2), a crucial component of LDs as well as significantly interrupted lactate-induced LD accumulation in prostate tumor cells and reduced growth and metastasis to the lungs in a prostate cancer xenograft mouse model [95]. Overall, LDs in tumor cells not only act as a promoter to provide essential energy for cell proliferation and invasion, but also serve as a protector against all external stresses that prevent lipotoxic cell damage and engage in a complex relationship with autophagy [84, 96].

## Functional differences between DGAT1 and DGAT2 in tumors

DGAT1 and DGAT2 are encoded by different gene families in humans [97]. The DGAT1 gene has been mapped to chromosome 8q24.3, and the encoded region is approximately 500 amino acids in length with 10 predicted transmembrane domains (TMDs) and presents homology with the monoacylglycerol acyltransferase gene [29, 97]. The DGAT2 gene belongs to an evolutionarily conserved acyltransferase gene family, located at chromosome 11q13.5. As an integral membrane protein, DGAT2 consists of 320 amino acids with 2 predicted TMDs, with additional acyl-CoA retinol acyltransferase activity [31, 97, 98]. Since the two DGATs belong to unrelated protein families, DGAT1 and DGAT2 differ in their substrate affinities, topology, protein partners, and cellular functions, and DGAT1 and DGAT2 have played non-redundant roles in TG synthesis in different tissues and species (Table 1) [71]. Furthermore, DGAT1 is proposed to be more focused on lipogenesis with exogenous FAs or FAs released by lipolysis and appears to have a broader substrate specificity (with respect to acyl acceptors) than DGAT2, while DGAT2 does not exhibit a propensity for substrates or prefers to use de novo synthesized FAs to synthesize TGs [39, 85, 86, 99–101]. Other than DGAT1, DGAT2 is responsible for most of the TG synthesis, which is essential for post-natal survival in mouse models [102]. DGAT2−/− mice die within hours of birth due to lack of a wax-ester dependent water-barrier in their skin, accompanied by profound reductions in systemic TGs [31]. Feeding C57BL/6 mice with 4 weeks high-fat diet significantly increased mouse body weight compared to a control group, and the expression of DGAT2 increased by 1.9-fold in white adipocytes, while DGAT1 decreased by 0.73-fold compared to that in mice fed a standard diet. DGAT1 knockout mice were viable, generally lean, more leptin- and insulin-sensitive, and resistant to high-fat diet-induced obesity [32].

**Table 1:** Differences between DGAT1 and DGAT2

	DGAT1	DGAT2
Subcellular localization	ER membrane	ER membrane and surface of LDs [168]
Substrate affinities	Exogenous FAs	No property or prefer to use DNL FAs
Topology	N terminus oriented toward the cytoplasmic matrix and C-terminal present in the ER lumen [169]	Both the N and C termini oriented toward the cytosol [169]
Highest expression tissue in human	Small intestine	Adipose tissue
Phenotypic consequences when deficient in mice	Modest reductions in tissue TG and lean phenotype [168]	Severe decreases in whole-body TG (90%) and die soon after birth [112]
Formation of LDs	Small LDs around the cell periphery [170]	Large cytosolic LDs [112]
Additional acyltransferase activities	Monoacylglycerol acyltransferase (MGAT), wax monoester and wax diester synthetases	Acyl-CoA retinol acyltransferase (ARAT) [35]

**Fig. 3:**
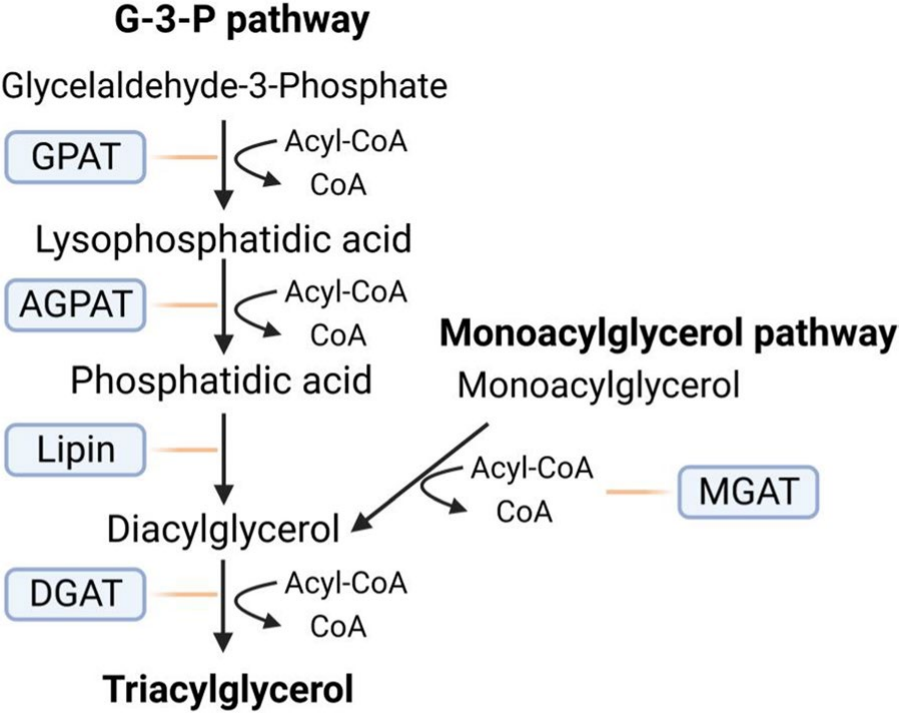
The synthesis of TGs in cancer cells. TG is synthesized by two major biochemical pathways, i.e., the glycerol 3 phosphate pathway (G3P) and the monoacylglycerol (MAG) pathway. In the G3P pathway, fatty acyl-CoA was condensed with G3P backbone to synthesize phosphatidic acid under the catalyzation of the glycerol-3-phosphate acyltransferase (GPAT) and acylglycerolphosphate acyltransferase (AGPAT), followed by dephosphorylation to obtain diacylglyceride (DAG). Further, DAG is converted to TG under the catalyzation of DGAT1 and DGAT2 [165]. In the MAG pathway, monoacylglycerol acyltransferases (MGATs), an isoform of which includes MGAT 1–3 located in the endoplasmic reticulum (ER) membrane, are the main enzymes responsible for TG synthesis that catalyze conversion of MAG to DAG along with fatty acyl-CoA [66, 67]. After synthesis of DAG, DGAT1 and DGAT2 are responsible for the synthesis of TG by using DAG and fatty acyl-CoA as substrates

In human beings, DGAT1 and DGAT2 are ubiquitously expressed but are most abundant in tissues involved in TG metabolism. In particular, DGAT1 is highly expressed in the small intestine and duodenum, and DGAT2 is highly expressed in adipocytes [103]. An analysis of the cancer genome atlas (TCGA) database showed that an elevated mRNA level of DGAT1 was observed in ovarian, lung, gastric, prostate, breast, liver, head and neck, melanoma, pancreas, sarcoma, cervical, thymoma, thyroid, and renal cancers, while the DGAT2 mRNA level was upregulated in bladder, breast, head and neck, and thyroid cancers [104, 105]. Indeed, the function of either DGAT1 or DGAT2 will be increased to compensate if the other is functionally deficient. For instance, mice lacking either DGAT1 or DGAT2 in adipocytes showed no differences in TG storage, suggesting that each enzyme can compensate for the lack of the other [106]. Likewise, DGAT1 deficiency showed no effect on energy and glucose metabolism in leptin-deficient mice due to a compensatory upregulation of DGAT2 expression in the absence of leptin [107, 108]. In contrast, despite both DGAT1 and DGAT2 being highly expressed in the liver, they seem to have distinct functions in the synthesis of TGs in hepatocytes. Disruption of DGAT1 or DGAT2 resulted in a marked reduction of TG levels in the liver [107, 109]. Further studies demonstrated that TGs produced by DGAT1 appear to be preferentially utilized for the oxidation of FAs, whereas TGs produced by DGAT2 are used for very low-density lipoproteins [110, 111]. Even with normal DGAT1 activity, DGAT1 was unable to completely compensate for the absence of DGAT2 and reverse death in DGAT2 deficient mice, underlying the different roles of the two DGATs in vivo [107, 108, 112]. Pathologically, decreased DGAT2 mRNA has been associated with the occurrence of psoriasis in human beings, and mutations in DGAT1 have been identified in a large cohort of patients with congenital diarrheal disorders [113, 114] (Fig. 3).

## Effects of activity of DGAT1 and DGAT2 on tumor cell proliferation

Tumor cells convert increased free FAs into cell-harmless neutral lipids and store them in LDs with the help of DGATs, thereby decreasing their further oxidation and protecting tumor cells from ROS and lipotoxicity [104, 115–117]. Accumulation of LDs is prevalent in a variety of tumor cells, especially those of patients who are obese [3, 14, 118, 119]. The expression of DGAT1 in glioblastomas (GBM) tissues was significantly higher than that of DGAT2, and the expression of DGAT1 in poorly differentiated tumor tissues was higher than that in well-differentiated tumor tissues [104]. Overexpression of DGAT1 in GBM cells facilitated cell proliferation by preventing oxidative stress via channeling excess FAs into TGs and LDs [120]. Inhibiting DGAT1 using the small inhibitor A-922500 suppressed cell proliferation, led to upregulation of CPT1A protein, promoted FAO and production of ROS, and induced apoptosis in GBM cells [96, 104, 105]. DGAT1 is frequently upregulated in melanoma, and overexpression of DGAT1 has been shown to cooperate with oncogenic B-Raf proto-oncogene, serine/threonine kinase (BRAF) or neuroblastoma RAS viral oncogene homolog (NRAS) in p53 mutant melanocytes, resulting in more rapid melanoma formation. Inhibition of DGAT1 blocked the incorporation of FAs to TGs and effectively suppressed tumor growth by increasing ROS production in a time-dependent manner in multiple melanoma cell lines, leading to an increase in lipid peroxidation from 24 h, with further increases by 48 h both in cytoplasm and the mitochondria, specifically, and ferroptosis. Conversely, stable overexpression of DGAT1 was protective against ROS-mediated cell death triggered by chemical ROS inducers [121]. Furthermore, compared with peripheral blood mononuclear cells, the prostate cancer LNCaP cell line showed a higher expression level of DGAT1 and a lower expression level of DGAT2. Targeting DGAT1 by siRNA in LNCaP cells led to an approximately 50% reduction of cell viability and prevented 90–95% formation of cell colonies through induction of cell cycle G1 arrest and autophagy [122]. In addition to decreasing the content of LDs, the DGAT1 inhibitor reduced the number and stability of the non-centrosomal microtubule-organizing center and decreased the levels of GM130, the CLIP-associated proteins 2 (CLASP2), and γ-tubulin in prostate tumor cells, thereby resulting in inhibition of cell proliferation and tumor growth [116].

Treatment of DGAT2-high-expressing MCF-7 cells with a DGAT2 inhibitor (PF-06424439) reduced the LD formation and cell proliferation through induction of cell cycle G2/M arrest [123]. When PF-06424439 was combined with radiation (6 Gy) to treat MCF cells, the combination treatment significantly increased the expression of H2A histone family, member X (H2Ax) compared with monotherapy, suggesting that inhibition of DGAT2 increases radiation-induced DNA damage [35, 123, 124] (Table 2). However, overexpression of DGAT2 by genetic modification in the hepatocellular carcinoma (HCC) cell lines, Hep3B and Huh7, resulted in inhibition of cell proliferation, reduction of colony formation, and induction of cell cycle arrest in G1 phase, while having no effect on apoptosis. After BALB/c nude mice were injected with DGAT2-overexpressing HCC cells for 28 days, the tumor weight and volume were markedly decreased compared with the control group [125]. The reason why high expression of DGAT2 appears to inhibit tumor growth in HCC remains unclear. One possible explanation is that FA metabolism is extremely active in hepatocytes, and the increased DGAT2 activity with lower free FAs and higher TG synthesis may not be favorable to tumor growth in HCC.

**Table 2:** Effects of DGAT1/2 inhibitors on tumor cell growth

Type of tumor	Inhibition of DGATs	Effects on tumor cell growth
Glioblastoma	DGAT1 inhibitor (A-922500)	Suppressed cell proliferation. Induced cell apoptosis and upregulation of CPT1A protein [96, 104, 105]
	Inhibition of DGAT1 by shRNA or miRNA-3918	Increased sensitivity of irradiation and prolonged survival of mice [141]
Melanoma	DGAT1 inhibitors (AZD3988, AZD7687, A922500, or T863)	Suppressed cell proliferation and cell cycle progression. Increased the expression of cleaved caspase-3 protein [121]
Gastric cancer	DGAT1 inhibitor (A-922500)	Reduced cell proliferation. Induced apoptosis [147]
Prostate cancer	Inhibition of DGAT1 by siRNA or A-922500	Reduced cell proliferation. Induced of cell cycle G1 arrest and autophagy [122] [116]
Breast cancer (MCF-7 cells)	DGAT2 inhibitor (PF-06424439)	Inhibited cell proliferation. Induced cell cycle G2/M arrest. Increased sensitivity of radiation. Reduced cell invasive ability. Reduced LD formation [35, 123, 124] Increased the production of ROS and unrepaired DNA damage of tumor cells after irradiation [123]
Breast cancer (MDA-231 cells)	DGAT1 inhibitor (A-922500)	Reduced cell proliferation and migratory ability [124]
Ovarian cancer	Knockdown of DGAT1	Inhibited cell proliferation. Reduced cell migration through modulating the EMT process [38]
Gastric cancer	Knockdown of DGAT2	Reduced mesenteric metastatic nodules in the intestinal wall and lung metastasis [39]
Colon cancer	DGAT1 inhibitor (A922500) and DGAT2 inhibitor (PF-06424439)	Inhibition of DGAT1 and DGAT2 decreased tumor growth and reduced the proportion of CD206 + MHCII ^low^ immunosuppressive myeloid cells in the tumors [150]

## Effects of activity of DGAT1 and DGAT2 on invasion and metastasis in tumor cells

Cervical cancer with pelvic lymph node metastasis has a poor prognosis, and higher LD content accompanied by elevated FASN expression in tumor tissues has been positively related to the lymph node metastasis rate in a mouse model of cervical cancer [126]. Inhibiting LD accumulation by a specific FASN inhibitor in a xenograft lymph node metastasis mouse model of cervical cancer significantly decreased tumor size and contributed to a lower incidence of lymph node metastasis [127]. Mechanistically, miR-532-5p may be involved in the regulation of LD formation in cervical tumor cells by bounding to LINC01410 and modulating the downstream FASN activity [127, 128]. Inhibition of DGAT1 activity by a DGAT1 inhibitor (A922500) significantly reduced TG synthesis, cell proliferation, and the migratory ability of breast cancer MDA-231 cells, accompanied by decreased expression levels of cyclin D1 and Zeb1 [124]. Similarly, knockdown of DGAT1 in the ovarian cancer OVCAR-5 and PEO4 cell lines inhibited cell proliferation, colonization, and migration. Results of RNA sequencing revealed that DGAT1 inhibited cell invasion by partly modulating the epithelial-mesenchymal transition (EMT) through the Wnt/β-catenin signaling pathways in ovarian tumor cells [38].

Metastasis to the omentum or peritoneum occurs in more than 50% of advanced gastric cancer cases, leading to a five-year overall survival rate of less than 7% [129]. Anoikis is a kind of intrinsic apoptosis initiated by detachment from the extracellular matrix and characterized by decreased absorption of glucose and increased production of ROS, resistance of which is essential during cancer metastasis [130]. Increased DGAT2 activity was detected in gastric tumor cell lines when co-cultured with adipocytes, which subsequently upregulated the FAO pathway and production of NADPH and decreased intracellular ROS, thereby promoting the resistance of tumor cells to anoikis [123]. Knockdown of DGAT2 significantly reduced mesenteric metastatic nodules in the intestinal wall and lung metastasis in a gastric cancer lung metastasis nude mouse model, compared to a control group [39]. Consistently, inhibiting DGAT2 activity by a DGAT2 inhibitor (PF-06424439) effectively reduced the content of LDs and cell invasion ability in breast cancer MCF-7 cells, increased expression of E-cadherin, and suppressed expression of Vimentin and Snail [123]. These results suggest that the inhibition of DGATs suppressed tumor cell invasion and metastasis partly by modulating the EMT process (Table 2).

## Effects of activities of DGATs on the response of tumor cells to chemotherapy and radiotherapy

Chemoresistance is a major challenge in the treatment of patients with metastatic or recurrent cancer, either because the initial tumor fails to respond to the treatment or because it acquires resistance during relapse [131]. Given that LDs are important intracellular organelles that buffer various intracellular stimuli, including cellular damage caused by excessive FAs and cell stress, accumulation of LDs has been proposed to contribute to the development of the drug-resistant phenotype of tumor cells, by neutralizing cytotoxicity induced by anti-tumor drugs and by impairing the activation of caspase cascades and ER stress responses [81, 132–134]. Supplementation of 1% oleic acid-Albumin from bovine serum to cervical cancer HeLa cells increased the LD content by three- to fourfold, and the potency of doxorubicin against the HeLa cells was considerably reduced in an oleic acid treatment group compared to the control group (IC_50_ > 1uM vs = 90 nM). The underlying mechanism of this observed effect may be that the LD hydrophobic core offers a compartment capable of attracting and sequestering lipophilic compounds and lipophilic drugs, thereby inhibiting their cytotoxic effect [135, 136]. Likewise, reducing the content of LDs by silencing lysophosphatidylcholine acyltransferase 2 sensitized colorectal cancer HT29 cells and SW620 cells to 5-fluorouracil and oxaliplatin, and similar results were also observed in a colorectal cancer xenograft mouse model [81]. Accumulation of LDs was observed in sorafenib-resistant HCC HepG2R and Huh7R cells compared to their parental HCC cells, and supplementation with AKR1C3 inhibitors to suppress the formation of LDs combined with sorafenib in HepG2R and Huh7R cells effectively increased the level of ROS and activated cell apoptosis [133, 137]. Indeed, the intrinsic presence of LDs has been widely reported to be a characteristic of chemoresistance in tumor cell lines, and a positive relation between LD content and tumor cell stemness has been demonstrated in different types of cancers, including ovarian, breast, colorectal, and pancreatic cancer [3, 134, 138, 139]. Thus, LD accumulation in tumor cells has been considered a critical bridge that triggers chemoresistance for tumor cells. Although there is still a lack of research on the relationship between the activity of DGATs and chemoresistance in tumor cells, the potential for targeting DGATs in tumor cells to reduce chemoresistance is of great interest, considering the key role of DGATs in the synthesis of TGs and LDs.

Metabolic advantages in tumor cells can promote the repair of ionizing radiation-induced DNA strand breaks and minimize the irradiation cytotoxicity to help tumor cells survive radiotherapy, contributing to cancer progression and recurrence [140]. Radiation was proposed to induce upregulation of DGAT1 and the formation of LDs in GBM U87MG cells, especially in radioresistance U87MG-RR cells, which showed a nearly 2.5-fold increase in the DGAT1 mRNA level compared to control cells. Inhibition of DGAT1 by using a short hairpin RNA (shRNA) re-sensitized the effects of radiation on cell proliferation and cellular stress in radioresistant GBM cells through attenuating FAO and protecting against mitochondrial lipotoxicity. When combined with radiation, inhibition of DGAT1 by shRNA or miRNA-3918 in a xenograft mouse model of GBM U87MG radioresistance significantly attenuated tumor size and suppressed tumor growth by 65.34% and 53.64%, respectively, and prolonged survival of the mice for nearly 10 days compared with radiation alone (2 Gy, 5 times) through induction of apoptosis [141]. The peroxidation of excess FAs after inhibiting DGAT2 by PF-06424439 further increased the level of ROS and reactive nitrogen species induced by irradiation in breast cancer MCF-7 cells, causing more severe and unrepaired DNA damage [123]. Pre-treatment of MCF-7 cells with PF-06424439 followed by irradiation reduced the transcript levels of CD44 and CD166, two well-recognized CSC markers commonly expressed among primary breast carcinomas, compared to radiation without PF-06424439, suggesting that activity of DGAT2 may be involved in the regulation of CSC [123] (Table 2).

## Effects of activity of DGAT1 and DGAT2 on antitumor immunity

Increasing evidence demonstrates that the aberrant tumor microenvironment induced by reprogramming the metabolism of tumor cells and the metabolites or intermediates of metabolism serves to regulate the proliferation, differentiation, and activation of host immune cells [142]. Accumulation of LDs was observed in various immune cells in cancer tissues, including T cells, dendritic cells (DCs), macrophages, mast cells, and neutrophils, which may be one of the characteristics of immune cells in a tumor microenvironment (TME) [20, 143–145]. As a marker of LD, PLIN2 was highly expressed in tumor-infiltrating immunocytes of oral squamous cell carcinoma, and higher PLIN2 presentation in the immunocytes effectively induced immune suppression characterized by less infiltration of CD8+ T cells and more CD68+ tumor-associated macrophages (TAMs) and Foxp3+ Tregs, with more immune checkpoint molecules such as CSF1R, homo sapiens galectin 9 (LGALS9), interleukin-10 (IL-10), cytotoxic T lymphocyte-associated antigen-4 (CTLA-4), and T-cell immunoglobulin and ITIM domain proteins (TIGIT). Patients with oral squamous cell carcinoma at a late TNM stage exhibited higher levels of PLIN2 in immune cells and were susceptible to postoperative metastasis, indicating that LDs may regulate host immunity by affecting the function of immune cells [146]. Overexpression of DGAT1 in both gastric cancer cells and tumor-infiltrating macrophages of patients with gastric cancer compared to normal tissues was associated with poor overall survival in gastric cancer patients. Inhibiting DGAT1 activity by a DGAT1 inhibitor (A922500) significantly increased early and late apoptosis of gastric cancer MKN45 cells in the presence of sodium oleate compared to A922500 alone [147]. Co-culturing DCs with tumor explant supernatants increased the level of lipids in DCs by threefold compared to DCs cultured in a control culture medium. Pre-incubation of DCs with neutralizing antibodies to Msr1 (CD204) for 72 h completely abrogated the effects of tumor explant supernatants on lipid accumulation, whereas the CD36-specific antibody had little effect on lipid accumulation of DCs, indicating that upregulation of Msr1 plays a major role in accumulation of lipids in DCs in cancer. DCs with an increased level of lipid had substantially lower stimulatory activity on allogeneic T-cell proliferation in a CT26 tumor-bearing mouse model compared to DCs from control mice. A lower response of OT-II T cells was observed in DCs with an increased level of lipid compared to DCs with normal lipid levels in EG-7 tumor bearing mice, suggesting that DCs with an increased level of lipid may have defects in processing tumor-associated proteins [148]. TAMs exert pro-tumoral properties when differentiated as M2-like macrophages, and the infiltration of M2-like macrophages is positively related to all stages of tumor progression [149]. Accumulation of LDs in TAMs was essential to induce the polarization of TAMs and subsequently promoted the immunosuppressive phenotype of TAMs (M2-like) that facilitate tumor growth by regulating the catabolism of free FAs for mitochondrial respiration via the mTOR pathway in colon tumor cells. Disrupting the formation of LDs by a DGAT1 inhibitor (A922500) and DGAT2 inhibitor (PF‐06424439) impeded tumor growth in a xenograft mouse model of colon cancer, decreased the proportion of CD206^+^MHCII ^low^ immunosuppressive myeloid cells in the tumors, and enhanced the effects of anti-tumor immunity [150] (Table 2). Another in vivo study also proposed that xenograft tumors induced by LDs-enriched colon cancer CT26 cells were twice as large as those induced by CT26 with low LDs. Furthermore, tumor tissues induced by CT26 cells enriched in LDs were detected with lower CD8+ T cells infiltration [81]. Thus, modulating the lipid storage process of tumor-associated immune cells may be an effective way to regulate anti-tumor immunity.

## Relationship of the expression of DGAT1 and DGAT2 and the prognosis of cancer patients

A controversial association between the prognosis of cancer patients and the expression levels of DGAT1 and DGAT2 in different types of tumors has been reported [119, 125, 147]. DGAT1 expression was increased in stage IV ovarian cancer compared with stage III ovarian cancer, and overexpression of DGAT1 was more common in poorly differentiated ovarian cancer compared with well-differentiated ovarian cancer [38]. Consistently, overexpression of DGAT1 in gastric cancer tissues was identified compared to normal tissues, and gastric cancer patients with overexpression of DGAT1 (n = 109) had a lower overall survival rate (13.7%) after 50 months follow-up compared to patients with low expression of DGAT1 (n = 56, 39.3%) [147]. The expression level of DGAT1 and LD content in GBM tumor tissues from patients with grade I-IV astrocytomas (n = 62) was detected using tissue microarray and immunofluorescence. Results showed that grade IV GBM tissues contained the highest levels of DGAT1 compared with anaplastic astrocytoma (AA, grade III), astrocytoma II (A2), and pilocytic astrocytoma (PA, grade I). The survival rate of GBM patients with high expression of DGAT1 (n = 58) was approximately 20% at 20 months follow-up, while patients with low DGAT1 expression (n = 97) showed about a 30% survival rate [104].

However, in lung adenocarcinoma, the mRNA level of DGAT1 was upregulated compared to normal tissues, and patients with high DGAT1 expression (n = 357) had a better survival rate compared to those with low DGAT1 [151]. Overexpression of DGAT2 in the tumor tissues predicted longer survival in patients with HCC (70% vs 50%, p = 0.02) after nearly five years of follow-up [125]. In addition, both DGAT1 and DGAT2 were upregulated in 179 pancreatic cancer tissues compared to 171 normal pancreatic tissues; however, pancreatic cancer patients with high or low expressions of DGAT1 or DGAT2showed no significant difference in overall survival after five years follow-up [119]. Collectively, the clinical significance of DGATs in prognosis may be revealed more clearly with a better understanding of the mechanism of regulation of DGAT on lipid metabolism in different types of tumors.

## Clinical application and limitations of inhibitors of DGAT1 and DGAT2

Since accumulation of excess TGs can lead to the pathogenesis of multiple metabolic disorders, such as obesity, type II diabetes, and fatty liver disease, DGAT1 and DGAT2 have been considered potential targets for the treatment of metabolic diseases [152]. Several compounds isolated from plants, microorganisms, and fish oils have been reported to selectively inhibit the activity of DGAT1 or DGAT2 with potent inhibitory effects on the synthesis of TGs in mammal cells [71, 153]. Meanwhile, various small molecule inhibitors for DGATs from multiple chemical series have been developed since the last decade. These DGAT1 or DGAT2 inhibitors have effects on metabolism similar to those observed in cells or mice with knockout of DGAT1 or DGAT2 [154–156]. Currently, clinical trials of DGAT1 and DGAT2 inhibitors for obesity, diabetes, and other metabolic disorders are being tested as a single agent or in combination with another metabolic regulatory drugs, such as acetyl-CoA carboxylase inhibitors (Table 3) [157, 158].

**Table 3:** DGAT inhibitors in clinical trials

	Target	Clinical trial	Disease	Primary outcome
PF-04620110	DGAT1	Phase I NCT01064492	12 healthy volunteers	
		Phase IB NCT01298518	48 type 2 diabetes mellitus subjects	Reduced postprandial TG excursions [159]
AZD7687	DGAT1	Phase I NCT01046357	80 healthy male subjects	Decreased postprandial TG excursions approximately 75% compared to placebo following a fat containing meal (p < 0.0001) [160]
Pradigastat (LCQ-908)	DGAT1	Phase I [172]	Overweight or obese healthy subjects (single-dose cohorts, n = 72) (multiple-dose cohorts, n = 106)	Pradigastat treatment (single and multiple doses) led to dose-dependent suppression of postprandial triglyceride excursions over 9 h following a high-fat meal test. Pradigastat was safe and tolerated at single and multiple doses in healthy subjects
		Phase III NCT01514461	45 adults with familial chylomicronemia syndrome (FCS) (Hyperlipoproteinemia type I)	Reduced both postprandial and fasting triglycerides from baseline to 12 weeks after treatment (fasting TG: LCQ908 vs placebo: − 13.9 vs 45.6, p = 0.0182)
Ervogastat (PF-06865571)	DGAT2	Phase I NCT04044053	12 healthy adult participants	
		Phase I NCT03092232	17 healthy adult participants	
		Phase I NCT03230383	60 healthy adults including overweight and obese	
		Phase I NCT03513588	48 people with nonalcoholic fatty liver disease	Relative change from baseline in whole liver fat at Day 15 after treatment (PF-06865571 300 mg vs placebo: − 41.14 vs − 10.94, p < 0.001)
		Phase II NCT04399538	75 adult participants with presumed nonalcoholic steatohepatitis	DGAT2i combined with ACC inhibitors significantly reduced percentage of liver fat after 6 weeks treatment compared to placebo (− 58.80% vs − 3.58, p < 0.001)
		Phase II NCT04321031	450 non-alcoholic steatohepatitis with fibrosis	

Thus far, at least three DGAT1 inhibitors have been advanced into clinical studies. PF-04620110 was the first pharmacological inhibitor of DGAT1 tested in healthy humans and patients with type 2 diabetes, and this drug demonstrated a dose-dependent reduction of postprandial TG excursions with varying degrees of gastrointestinal adverse effects (NCT01298518) [159]. AZD7687 is a novel potent, selective, and reversible small molecule inhibitor of DGAT1. Oral supplementation of AZD7687 in healthy individuals effectively decreased the serum TG excursion following a high-fat meal by reducing the delivery of TGs and chylomicrons from the gut. However, dose-limiting gastrointestinal adverse events, including nausea, diarrhea, and vomiting, were observed, especially when following a high-fat diet [160]. Similarly, another phase I clinical trial of AZD7687 in 62 overweight or obese men exhibited dose-dependent reductions in postprandial serum TG and an elevation of glucagon-like peptide-1 and peptide YY levels when treated with AZD7687 ≥ 5mg compared with a placebo (p < 0.01). However, gastrointestinal side effects of AZD7687, particularly diarrhea, increased with AZD7687 doses of > 5 mg/day, making the utility of AZD7687 as a novel treatment for diabetes and obesity questionable [161]. Pradigastat (LCQ-908), a specific DGAT1 inhibitor, has also been shown to decrease postprandial TG either administered at single or multiple doses following a high-fat diet as well as reduce postprandial glucose and increase plasma glucagon-like peptide-1 levels in overweight or obese healthy individuals [162]. Orally administered pradigastat effectively reduced both postprandial and fasting TG level in patients with FCS in a phase III clinical trial. Patients with FCS seemed to tolerate pradigastat as a low-fat diet was used as a standard of care for these patients and reduced the severity of diarrhea (NCT01514461). The dose-limiting gastrointestinal adverse events observed in these clinical trials indicate that it is difficult to achieve a substantial level of therapeutic drug exposure with oral administration, especially when facing a high-fat diet challenge [163].

Multiple differing characteristics between DGAT1 and DGAT2, including gene family, subcellular localization, substrate preference, and specific expression pattern in different organs, results in unrelated pharmacologic inhibition properties and distinct safety profiles of DGAT1 and DGAT2 inhibitors [164]. Unlike DGAT1, DGAT2 inhibitors have not been initially pursued as aggressively as a potential target for pharmacologic intervention. Ervogastat (PF-06865571), a novel, potent, and specific DGAT2 inhibitor, has shown safety and promise in reducing hepatic steatosis in early clinical trials (NCT03230383, NCT03513588). In a phase II clinical trial of presumed nonalcoholic steatohepatitis (NASH), Ervogastat effectively reduced the percentage of liver fat by 58.80% compared to baseline before treatment, accompanied by a well-tolerated safety profile (NCT04399538) (ClinicalTrials.gov, EudraCT, and/or www.pfizer.com). Another larger scaled phase II clinical trial of Ervogastat with NASH is ongoing, aiming to assess the efficacy and safety of the inhibition of DGAT2 and ACC to resolve NASH with fibrosis (NCT04321031) [165].

Despite anti-tumor effects reported for DGAT inhibitors in pre-clinical cancer models, the side effects of DGAT inhibitors, especially gastrointestinal adverse events, were observed in finished clinical trials, and ubiquitous expression of DGATs in the whole body and an unsatisfactory pharmacokinetic profile may partially limit the application of current inhibitors of DGAT1 and DGAT2 in cancer patients [166, 167]. It is expected that new and improved small molecular inhibitors of DGATs will be used in future clinical trials, including those for cancer patients [165].

## Conclusion

As the crucial puzzle pieces that trigger LD formation, DGATs are greatly involved in the regulation of tumor cell proliferation, invasion, anti-tumor immunity, and response to chemotherapy and radiotherapy in tumor cells and mouse models. By reprogramming the activities of DGAT1 and DGAT2, tumor cells can form more LDs to tolerate their enhanced free FA metabolism and prevent possible lipotoxicity and cell death. Notably, different roles of DGAT1 and DGAT2 in tumor growth and prognosis have been reported in different types of tumors, increasing the difficulty of applications of DGATs in the clinical setting. Several inhibitors of DGAT1 and DGAT2 have been created over the past decade, showing anti-tumor effects in pre-clinical models of tumors and metabolic improvements in clinical trials. Side effects of existing inhibitors of DGATs, especially gastrointestinal adverse events, have limited the further exploration of DGAT inhibitors in cancer patients. Further identification of the function of DGATs in cancer cell growth, especially the mechanisms regulating tumor growth in different tumor types, will certainly help to better understand the potential role of DGATs in tumor treatment and prognosis.

## Data Availability

Data sharing is not applicable to this article as no datasets were generated or analyzed during the current study.

## Abbreviations

ACLY: ATP-citrate lyase
ACC: Acetyl-CoA carboxylase
ACS: Acyl-CoA synthetase
ACAT: Acyl-CoA:cholesterol O-acyltransferases
AGPAT: Acylglycerolphosphate acyltransferase
CSC: Cancer stem cell
CAF: Cancer-associated fibroblast
DAG: Diacylglyceride
DGAT: Diacylglycerol acyltransferase
DNL: De novo lipogenesis
DC: Dendritic cell
ER: Endoplasmic reticulum
EMT: Epithelial-mesenchymal transition
FAO: β-Oxidation of FAs
FASN: Fatty acid synthase
FA: Fatty acid
FAS: Fatty acid synthesis
FABPpm: Plasma membrane fatty acid-binding proteins
FATPs: Fatty acid transport protein family
FAT: Fatty acid translocase
G3P: Glycerol-3-phosphate
GPAT: Glycerol-3-phosphate acyltransferase
GBM: Glioblastoma
HCC: Hepatocellular carcinoma
HSL: Hormone-sensitive lipase
KRAS: Kirsten rat sarcoma viral oncogene
LD: Lipid droplet
MAG: Monoacylglycerol
MGATs: Monoacylglycerol acyltransferases
NAFLD: Nonalcoholic fatty liver disease
NASH: Nonalcoholic steatohepatitis
OS: Overall survival
PTEN: Phosphatase and tensin homolog deleted on chromosome ten
PLIN: Perilipin
PAP: Phosphatidic acid phosphohydrolase
ROS: Reactive oxygen species
SCD1: Stearoyl-CoA desaturase-1
SE: Steryl ester
TMD: Transmembrane domain
TG: Triglyceride
TME: Tumor microenvironment
TAM: Tumor-associated macrophage
